# Arene Substitutions in Orchid Bibenzyls: Mechanistic Insights into Glucose Uptake and Lipid Metabolism for Targeting Metabolic Disorders

**DOI:** 10.3390/nu17071104

**Published:** 2025-03-21

**Authors:** Narawat Nuamnaichati, Utid Suriya, Hnin Ei Ei Khine, Rungroch Sungthong, Poon Suwannamai, Boonchoo Sritularak, Eakachai Prompetchara, Chavee Laomeephol, Rosa Alduina, Chatchai Chaotham

**Affiliations:** 1 Department of Biochemistry and Microbiology, Faculty of Pharmaceutical Sciences, Chulalongkorn University, Bangkok 10330, Thailand; 2 Department of Biochemistry, Faculty of Science, Mahidol University, Bangkok 10400, Thailand; 3 Department of Biotechnology, Faculty of Science, Mahidol University, Bangkok 10400, Thailand; 4 Department of Pharmacognosy and Pharmaceutical Botany, Faculty of Pharmaceutical Sciences, Chulalongkorn University, Bangkok 10330, Thailand; 5 Center of Excellence in Natural Products for Ageing and Chronic Diseases, Faculty of Pharmaceutical Sciences, Chulalongkorn University, Bangkok 10330, Thailand; 6 Department of Laboratory Medicine, Faculty of Medicine, Chulalongkorn University, Bangkok 10330, Thailand; 7 Center of Excellence in Vaccine Research and Development (Chula Vaccine Research Center), Faculty of Medicine, Chulalongkorn University, Bangkok 10330, Thailand; 8 Department of Biological, Chemical and Pharmaceutical Sciences and Technologies (STEBICEF), University of Palermo, 90128 Palermo, Italy; 9 Center of Excellence in Preclinical Toxicity and Efficacy Assessment of Medicines and Chemicals, Chulalongkorn University, Bangkok 10330, Thailand

**Keywords:** bibenzyl, arene substitution, glucose uptake, cellular lipid metabolism, myotube, adipocyte

## Abstract

Background: Phytochemicals possess diverse therapeutic potential; however, the impact of arene substitutions on the pharmacological properties of the bibenzyl compounds batatasin III and gigantol, derived from *Dendrobium venustum*, remains unexplored. Objectives: This study examines how structural differences between these compounds affect cellular glucose uptake and lipid metabolism during adipocyte differentiation. Methods: The effects of both bibenzyl compounds on cytotoxicity and glucose uptake were assessed in mouse and human pre-adipocytes and rat skeletal muscle myoblasts using colorimetric assays. Lipid metabolism was evaluated through Oil Red O staining and quantification of triglyceride and glycerol levels, while protein and gene expression during adipocyte differentiation were analyzed via western blotting and RT-qPCR. Results: At the highest non-cytotoxic concentration (25 µM), gigantol significantly enhanced glucose uptake (up to 2-fold) under both basal and insulin-stimulated conditions, whereas batatasin III showed a similar effect only under basal conditions. Gigantol upregulated GLUT1 and GLUT4 in myotubes but downregulated them in adipocytes, whereas batatasin III had minimal impact on these transporters. Both compounds suppressed lipid accumulation in mouse and human adipocytes by decreasing intracellular triglyceride content and promoting extracellular glycerol release. However, batatasin III did not affect extracellular glycerol release during early adipocyte differentiation, as evidenced by the marked downregulation of key lipogenic proteins (PLIN1, LPL, FABP4) observed only with gigantol. Molecular docking analyses suggest that gigantol’s greater bioactivity may result from its higher number of arene substitutions. Conclusions: This study provides the first evidence that differences in arene substitutions among orchid-derived bibenzyls influence their pharmacological properties. Our findings support the strategic modification of natural products as a potential approach for managing metabolic disorders.

## 1. Introduction

Natural compounds exhibit extraordinary structural diversity and are systematically categorized into distinct chemical classes, including alkaloids, flavonoids, polyphenols, and polysaccharides [1,2]. These classes encompass a broad spectrum of bioactive molecules with significant applications in disease prevention and treatment [2]. Beyond variations in their core chemical frameworks, plants further diversify their metabolites through strategic substitution and relocation of functional groups, thereby modulating their bioactivities, pharmacokinetics, and therapeutic potential [3,4,5,6,7]. Such structural modifications are not random but are often evolutionarily optimized to improve bioavailability, metabolic stability, and target specificity [7,8,9].

Among these modifications, arene substitutions exert a profound influence on the pharmacological profiles of natural products by altering their molecular interactions, receptor-binding affinity, and bioavailability [10]. Substitutions involving hydroxyl, methoxy, and halogen groups can significantly affect lipophilicity, hydrogen bonding capacity, and steric hindrance, collectively shaping the bioactivity profiles of these compounds [10,11,12,13,14,15,16]. Furthermore, positional substitutions on aromatic rings have been shown to enhance anticancer, anti-inflammatory, and antidiabetic properties by improving target specificity and minimizing off-target effects [17,18]. Computational docking and structure–activity relationship studies further reveal that these substitutions critically determine the binding affinity of natural products to key metabolic and signaling proteins, directly influencing their therapeutic efficacy [19,20].

Within this context, the genus *Dendrobium*, an extensive group of orchids widely employed in traditional Chinese medicine, has gained substantial recognition for its therapeutic potential in treating a variety of ailments, including dry mouth, fever, gastrointestinal disorders, diabetes, obesity, and associated complications [21,22]. These metabolic disorders—obesity, hyperlipidemia, insulin resistance, and diabetes mellitus—represent pressing global health concerns exacerbated by modern sedentary lifestyles and poor dietary habits [23,24]. Conventional pharmacological treatments often involve adverse side effects, driving increased interest in plant-based therapeutic alternatives as safer and more effective options.

Among the bioactive compounds derived from *Dendrobium*, bibenzyls represent a structurally intriguing and pharmacologically significant class of phytochemicals [18,25,26]. These compounds demonstrate a wide array of bioactivities, including antidiabetic, anti-inflammatory, antimicrobial, anti-obesity, antioxidant, anticancer, immunomodulatory, and neuroprotective effects [16,25,26]. Specific bibenzyl derivatives, such as amoenylin, 3,4-dihydroxy-5,4′-dimethoxybibenzyl, and 4,5,4′-trihydroxy-3,3′-dimethoxybibenzyl, have exhibited anti-obesity potential through the inhibition of adipocyte differentiation and lipid accumulation [25,26].

Structurally, bibenzyls consist of two phenyl groups bridged by an ethane moiety. These phytochemicals are biosynthesized through the integration of the shikimate and polyketide pathways, wherein phenylalanine undergoes a series of enzymatic transformations, ultimately yielding malonyl-CoA, which is then catalyzed by bibenzyl synthase to form the bibenzyl core structure [6,18]. Subsequent tailoring reactions further modify the bibenzyl backbone, contributing to its structural diversification. The pharmacological activities of bibenzyls are further refined by the substitution and positional rearrangement of functional groups, such as hydroxyl and methoxy groups on the aromatic rings [6,12,18]. These substitutions not only alter molecular polarity and chemical reactivity but also modulate protein–ligand interactions and cellular uptake mechanisms, highlighting the intricate relationship between structural variation and biological activity [6,12,18].

Despite substantial progress in understanding the pharmacological potential of bibenzyls, a significant knowledge gap remains regarding the specific effects of arene substitutions on their bioactivities. This lack of clarity limits the strategic optimization of these compounds for therapeutic applications.

In this study, we investigated the effects of two bibenzyl compounds isolated from *Dendrobium venustum*, batatasin III and gigantol, on glucose uptake and lipid accumulation in myotubes and adipocytes. Through comprehensive pharmacological analysis and mechanistic insights, this study aims to clarify the role of arene substitutions in modulating bioactivity. The findings are expected to provide valuable insights for optimizing the bioactivities of these pharmaceutical candidates, advancing their potential in drug development and contributing to the integration of plant-derived compounds into modern therapeutic strategies while preserving their historical and cultural significance.

## 2. Materials and Methods

### 2.1. Phytochemicals

**Figure 1:**
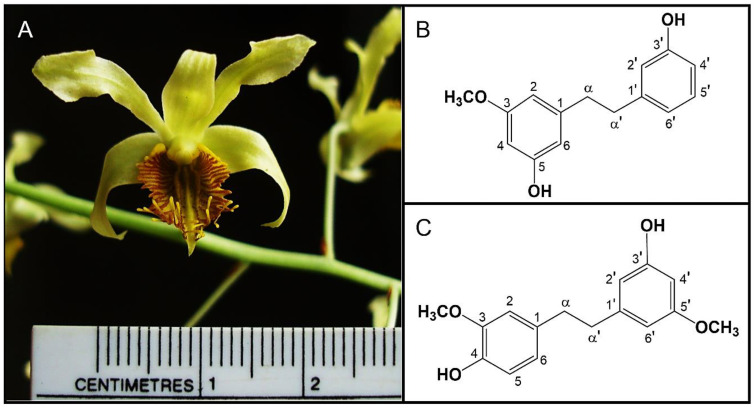
Two bibenzyl compounds used in this study. *Dendrobium venustum* (**A**), the natural source of both bibenzyls; batatasin III (**B**); and gigantol (**C**).

Batatasin III and gigantol were extracted from *D*. *venustum* using methanol as a primary solvent, followed by multi-step fractionation and purification processes (Figure 1). Their chemical identities were confirmed through mass spectrometry (MS) and nuclear magnetic resonance (NMR) spectroscopy in accordance with previously established protocols [27]. Both phytochemicals, each with a purity exceeding 98%, were stored at −20 °C under controlled conditions to ensure their stability and integrity for subsequent investigations.

### 2.2. Cell Cultures

All biological reagents, including Dulbecco’s Modified Eagle Medium (DMEM), were procured from Gibco^TM^ (Gaithersburg, MA, USA), while Fibroblast Basal Medium (FBM) was sourced from the American Type Culture Collection (ATCC, Manassas, VA, USA). Mouse 3T3-L1 pre-adipocytes, human PCS-210-010 pre-adipocytes, and rat skeletal muscle L6 myoblasts were obtained from ATCC. These cell models were selected for their relevance to key molecular pathways underlying targeted metabolic disorders, including diabetes and obesity. Among them, PCS-210-010 human adipocytes provide a clinically relevant system for assessing the pharmacological potential of emerging natural products in human metabolism. Cells were cultured in their respective complete growth media supplemented with 2 mM l-glutamine and 100 units mL^−1^ penicillin–streptomycin solution. Cell cultures were incubated at 37 °C in a humidified chamber with 5% CO_2_. DMEM supplemented with 10% fetal bovine serum (FBS) was used for culturing 3T3-L1 and L6 cells, while 2% FBS-supplemented FBM was employed for maintaining PCS-210-010 cells. Experimental procedures were conducted on cells that had reached 70–80% confluency to ensure optimal growth and physiological relevance.

### 2.3. Cell Differentiation Protocols

The adipocyte differentiation protocol followed in this study was previously described by Khine et al. [26]. In brief, monolayer cultures of 3T3-L1 and PCS-210-010 pre-adipocytes, cultured in complete medium, were allowed to reach 100% confluence before undergoing a series of medium replacements every two days. The differentiation process involved sequential exposure to differentiation medium (2 days), insulin medium (2 days), and complete medium (up to 8 days). The differentiation medium consisted of complete medium supplemented with 0.5 mM isobutylmethylxanthine, 1 µM dexamethasone (Sigma-Aldrich, St. Louis, MO, USA), and 5 µg mL^−1^ insulin (Himedia, Mumbai, India). The insulin medium was prepared by adding 5 µg mL^−1^ insulin to complete medium.

Differentiation was considered complete 8 days after the initial medium replacement, as indicated by the presence of intracellular lipid droplets, with an additional 2-day period required for full maturation into adipocytes. Experiments conducted on day 0 (the day differentiation medium was first applied) and day 8 (post-differentiation) corresponded to the early and late stages of differentiation, respectively.

For L6 myotube differentiation, a similar medium replacement approach was applied. L6 myoblasts were cultured in differentiation medium, composed of complete DMEM supplemented with 2% horse serum (Gibco^TM^, Gaithersburg, MA, USA). Medium was refreshed every 2 days over a minimum period of 8 days until the formation of multinucleated myotubes was observed, indicating successful differentiation.

### 2.4. Cytotoxicity Assay

All reagents and chemicals used in this experiment were ordered from Sigma-Aldrich (St. Louis, MO, USA). The cytotoxic effects of batatasin III and gigantol on both pre- and late-differentiated cells were evaluated using the 3-(4,5-dimethylthiazol-2-yl)-2,5-diphenyltetrazolium bromide (MTT) assay and dual nuclear staining method.

Pre-differentiated 3T3-L1 cells were seeded in 96-well plates containing complete medium and treated with varying concentrations (0–100 μM) of each phytochemical for 48 h. The non-cytotoxic concentrations were defined by showing no significant difference in cell viability compared to untreated vehicle controls supplemented with 0.5% (*v*/*v*) dimethyl sulfoxide (DMSO) solution. The chosen non-cytotoxic concentrations were also confirmed with other cell lines tested, and all of their late-differentiated cells, which were subjected to an 8-day differentiation protocol, underwent the same treatment procedure. Following 48 h of incubation in a 5% CO_2_-humidified incubator at 37 °C, the culture medium was replaced with 100 µL of 0.5 mg mL^−1^ MTT solution, and the cells were incubated in the dark at 37 °C for 3 h. Subsequently, the MTT solution was discarded, and 100 µL of DMSO was added to dissolve the formazan crystals formed by mitochondrial MTT reduction. Cell viability was quantified by measuring absorbance at 570 nm using a microplate reader (PerkinElmer, Waltham, MA, USA), with values expressed relative to vehicle control cells.

Cell death induced by each phytochemical was further examined using a dual nuclear staining approach with 2 µg mL^−1^ Hoechst 33342 and 1 µg mL^−1^ propidium iodide (PI) prepared in phosphate-buffered saline (PBS, pH 7.4). After 48 h of phytochemical treatment, cells were stained with the dye mixture for 30 min and visualized under a fluorescence microscope (Olympus IX51 with DP70, Tokyo, Japan). Dead cells were identified by bright blue fluorescence from Hoechst 33342 (indicating nuclear condensation or fragmentation) or red fluorescence from PI (indicating membrane permeability and cell death).

### 2.5. Glucose Uptake Assessment

The glucose uptake activity of differentiated cells treated with each phytochemical was assessed under both basal and insulin-stimulated conditions using the Glucose (GO) Assay Kit (Sigma-Aldrich, St. Louis, MO, USA). Following an 8-day differentiation protocol, cells were exposed to non-cytotoxic concentrations of each phytochemical for 48 h at 37 °C in a 5% CO_2_-humidified incubator. Treatments with 0.5% (*v*/*v*) DMSO and 1 mM metformin hydrochloride (Sigma-Aldrich, St. Louis, MO, USA) served as the vehicle control and positive control, respectively. For insulin-stimulated conditions, 100 nM insulin was added to each test group.

After the treatment period, culture media were collected, diluted with sterile distilled water, and analyzed for glucose levels. Each diluted sample (25 µL) was mixed with 50 µL of GO Assay Kit reagent and incubated at ambient temperature for 30 min. The reaction was then terminated by adding 50 µL of 6 M H_2_SO_4_ (Merck KGaA, Darmstadt, Germany). The glucose concentration was determined based on the absorbance measured at 540 nm using a microplate reader.

The relative glucose uptake was calculated using the following equation:

$$\mathrm{Relative}\;\mathrm{glucose}\;\mathrm{uptake}=\frac{{\mathrm{Abs}}_{\;\mathrm{blank}}-{\mathrm{Abs}}_{\;\mathrm{treatment}/\mathrm{positive}\;\mathrm{control}}}{{\mathrm{Abs}}_{\;\mathrm{blank}}-{\mathrm{Abs}\;}_{\mathrm{vehicle}}}$$

where Abs _blank_, Abs _treatment/positive control_, and Abs _vehicle_ refer to the absorbance value of the blank (fresh medium), absorbance value of the treatment or positive control group, and absorbance value of the vehicle control group, respectively. This calculation reflects the relative glucose uptake efficiency of cells treated with each phytochemical compared to the control groups under both basal and insulin-stimulated conditions.

### 2.6. Tracking Glucose Transporters

The translocation of glucose transporters in 3T3-L1 adipocytes and L6 myotubes was investigated following treatment with batatasin III or gigantol for 48 h at 37 °C. After treatment, cells were washed three times with ice-cold PBS and fixed with 4% formaldehyde (Sigma-Aldrich, St. Louis, MO, USA) for 10 min at ambient temperature. The fixed cells were rinsed three times with PBS and permeabilized using 0.1% Triton X-100 (Sigma-Aldrich, St. Louis, MO, USA) for 10 min at ambient temperature.

Subsequently, cells were blocked with 3% bovine serum albumin (Gibco^TM^, Gaithersburg, MA, USA) and incubated overnight at 4 °C with primary antibodies targeting glucose transporters GLUT1 (D3J3A Rabbit mAb) and GLUT4 (1F8 Mouse mAb), both obtained from Cell Signaling Technology (Danvers, MA, USA). After three additional washes with PBS, the cells were incubated for 2 h at ambient temperature in the dark with fluorescence-tagged secondary antibodies: Alexa Fluor™ 594 goat anti-rabbit IgG (H + L) for GLUT1 and Alexa Fluor™ 488 goat anti-mouse IgG (H + L) for GLUT4 (Cell Signaling Technology, Danvers, MA, USA).

Following the secondary antibody incubation, cells were washed three times with PBS and stained with Hoechst 33342 for 30 min at ambient temperature in the dark to visualize nuclei. Imaging was performed using a confocal microscope (Zeiss LSM 900 with Airyscan 2, Jena, Germany) to analyze the subcellular localization of GLUT1 and GLUT4.

### 2.7. Oil Red O Staining

The effects of batatasin III and gigantol on lipid accumulation in adipocytic cells were evaluated at both the early and late stages of differentiation using Oil Red O staining. Cells were treated with non-cytotoxic concentrations of each phytochemical on day 0 (early differentiation) and day 8 (late differentiation) of the differentiation protocol. Undifferentiated and differentiated vehicle control cells were included as experimental controls.

Following 48 h of treatment, cells were fixed with 10% formalin (Merck KGaA, Darmstadt, Germany) for 45 min at ambient temperature. The fixed cells were subsequently stained with Oil Red O dye (Sigma-Aldrich, St. Louis, MO, USA) for 30 min and then washed three times with water and 60% (*v*/*v*) isopropanol to remove excess dye. Stained cells were visualized and randomly imaged under an optical microscope (Nikon Ts2, Tokyo, Japan).

Quantification of Oil Red O content, representing lipid droplet accumulation, was performed using a colorimetric assay. Stained lipid droplets were eluted with 100% (*v*/*v*) isopropanol, and the absorbance of the resulting solution was measured at 510 nm using a microplate reader. The Oil Red O content was normalized to the total cellular protein level, determined using the Thermo Scientific Pierce BCA Protein Assay Kit (Rockford, IL, USA). Results were expressed as a percentage relative to the differentiated vehicle control group, providing a comparative measure of lipid accumulation across treatment conditions.

### 2.8. Intracellular Triglyceride and Extracellular Glycerol Measurements

Lipid metabolism in adipocytes during the differentiation program was tracked by measuring intracellular triglyceride and extracellular glycerol levels following 48 h treatments with phytochemicals at early and late differentiation stages. Undifferentiated and differentiated cells treated with 0.5% *v*/*v* DMSO were included as vehicle controls.

To quantify intracellular triglyceride content, treated cells were collected and subjected to extraction and purification using a biphasic chloroform/methanol mixture (2:1, *v*/*v*). The triglyceride concentrations in the extracts were determined using the Triglyceride Quantification Kit (Sigma-Aldrich, St. Louis, MO, USA), according to the manufacturer’s instruction, and correlated to a standard curve of known triglyceride concentrations.

For extracellular glycerol content, cell-free culture supernatants were collected and analyzed using the Glycerol Assay Kit (Sigma-Aldrich, St. Louis, MO, USA). Glycerol levels were quantified by correlating absorbance values to a standard curve of known glycerol concentrations, following the manufacturer’s protocol.

### 2.9. Cell Cycle Analysis

Cell cycle distribution, including the G0/G1, S, and G2/M phases, was evaluated using flow cytometry. Following a 48 h treatment with non-cytotoxic concentrations of batatasin III or gigantol, 3T3-L1 cells at the early stage of differentiation were collected and centrifuged at 2500× *g* for 5 min at 4 °C. The harvested cells were fixed in 70% ethanol (400 μL) for 2 h on ice and then washed twice with PBS (pH 7.4).

Subsequently, the fixed cells were incubated for 1 h at 37 °C in a staining solution containing 60 μg mL^−1^ RNase A (Thermo Scientific, Rockford, IL, USA) and 50 μg mL^−1^ propidium iodide (PI). Stained cells were analyzed using a Guava easyCyte flow cytometer (EMD Millipore, Austin, TX, USA) equipped with InCyte 3.3 software, and the resulting data were further processed using FlowJo™ V10 software (Becton, Dickinson and Company, Franklin Lakes, NJ, USA).

### 2.10. Western Blotting

The expression of proteins involved in the early and late differentiation of adipocytes treated with either batatasin III or gigantol for 48 h was evaluated using Western blotting. Briefly, treated cells were harvested for protein extraction, and the resulting cellular protein concentrations were normalized using the Thermo Scientific Pierce BCA Protein Assay Kit. Equal amounts of protein were resolved on 10% sodium dodecyl sulfate–polyacrylamide gel electrophoresis and transferred onto a nitrocellulose membrane (Bio-Rad Laboratories, Hercules, CA, USA).

The membrane was blocked with 5% skim milk (Sigma-Aldrich, St. Louis, MO, USA) prepared in Tris-buffered saline containing 0.1% Tween 20 (TBST, pH 7.4), and subsequently immunoblotted with primary antibodies specific to the target proteins. The primary antibodies included GLUT1, GLUT4, PPARγ, C/EBPα, FAS, FABP4, PLIN1, adiponectin, AKT, phosphorylated AKT (p-AKT; Ser473), AMPKα, phosphorylated AMPKα (p-AMPKα; Thr172), AMPKβ1/2, phosphorylated AMPKβ1 (p-AMPKβ1; Ser182), ACC, phosphorylated ACC (p-ACC; Ser79), GSK3β, phosphorylated GSK3β (p-GSK3β; Ser9), and β-Actin, all purchased from Cell Signaling Technology (Danvers, MA, USA), while LPL and SREBP-1c antibodies were sourced from Invitrogen (Waltham, MA, USA).

After three washes with TBST (~7 min per wash), the membranes were incubated with horseradish peroxidase-conjugated secondary antibodies (Cell Signaling Technology, Danvers, MA, USA). Protein bands were visualized using the Chemiluminescent ImageQuant LAS 4000 system (GE Healthcare Bio-Sciences AB, Uppsala, Sweden), and band intensities were quantified. The expression levels of each target protein were normalized to β-Actin (internal loading control) and reported as relative values.

### 2.11. Reverse Transcription-Quantification Polymerase Chain Reaction (RT-qPCR)

The transcriptional regulation of target genes was assessed in early differentiating adipocytes treated with batatasin III or gigantol for 48 h using reverse RT-qPCR. Total RNA was extracted and purified using the GENEzol™ Reagent (Geneaid, Taiwan) and converted into complementary DNA (cDNA) using the Thermo Scientific RevertAid™ First Strand cDNA Synthesis Kit (Rockford, IL, USA), following the manufacturer’s protocol.

For qPCR, 1 μL of the synthesized cDNA (equivalent to 50 ng) was used as a template in a reaction mixture containing 5 μL Bio-Rad Luna Universal qPCR Master Mix (Hercules, CA, USA), 10 μM forward and reverse primers (0.25 μL each, Supporting Information (SI) Table S1), and nuclease-free water to reach a final reaction volume of 10 μL.

Amplification was performed in a Bio-Rad CFX96 Touch Real-Time PCR Detection System (Hercules, CA, USA) with the following thermal cycling program: initial denaturation at 95 °C for 3 min followed by 40 cycles of denaturation at 95 °C for 5 s and annealing at 55 °C for 30 s. Quantification cycle (*C*_q_) values were recorded, and relative changes in gene expression were calculated using the 2^−ΔΔ*C*q^ method, with β-actin serving as the internal reference gene.

### 2.12. Molecular Docking Analysis

The three-dimensional (3D) structures of the selected target proteins—fatty acid synthase (FAS, PDB ID: 4PIV), lipoprotein lipase (LPL, PDB ID:6OB0), and fatty acid binding protein 4 (FABP4, PDB ID: 4NNT)—were retrieved from the Protein Data Bank (https://www.rcsb.org/, accessed on 1 December 2024). Molecular docking binding sites were defined based on the coordinates of the co-crystallized ligands, as detailed in SI Table S2. A docking box with uniform dimensions of 20 Å was applied to encapsulate the binding site.

The 3D molecular structures of batatasin III (PubChem ID: 10466989) and gigantol (PubChem ID: 3085362) were obtained from the PubChem database and verified using BIOVIA Discovery Studio Visualizer 2021 (Dassault Systèmes, Vélizy-Villacoublay, France). To validate the docking protocol, co-crystalized ligands were redocked into their original binding sites, and the resulting conformations were superimposed with the crystallized ligands for alignment accuracy.

Protein and ligand files were prepared in the pdbqt format using AutoDock Tools version 1.5.7, and all docking simulations were conducted using AutoDock Vina XB [28]. This approach allowed for a detailed assessment of binding interactions and affinities between the selected proteins and the compounds under investigation. However, all interactions analyzed in this study were derived from the docked poses, which do not account for dynamic conformational changes or solvation effects in protein–ligand binding. Therefore, the precise binding mechanisms underlying the inhibitory actions of these compounds require further investigation using molecular dynamics simulations and experimental validation.

### 2.13. Statistics

Data are expressed as means ± standard error of the means (SEMs) or standard deviations (SDs) and are derived from a minimum of three independent experiments. This sample size was chosen based on established biological research methodologies, ensuring result reproducibility and minimizing biological variability. While formal statistical power calculations were not performed, the selected sample size is consistent with standard practices in in vitro research, providing reliable biological insights. Statistical comparisons were conducted using one-way analysis of variance (ANOVA) followed by Tukey’s post hoc test, with a significance threshold set at *α* = 0.05. All statistical analyses were performed using GraphPad Prism version 8.0.2 (GraphPad Software, San Diego, CA, USA).

## 3. Results

### 3.1. Differential Effects of Batatasin III and Gigantol on Cellular Glucose Uptake

**Figure 2:**
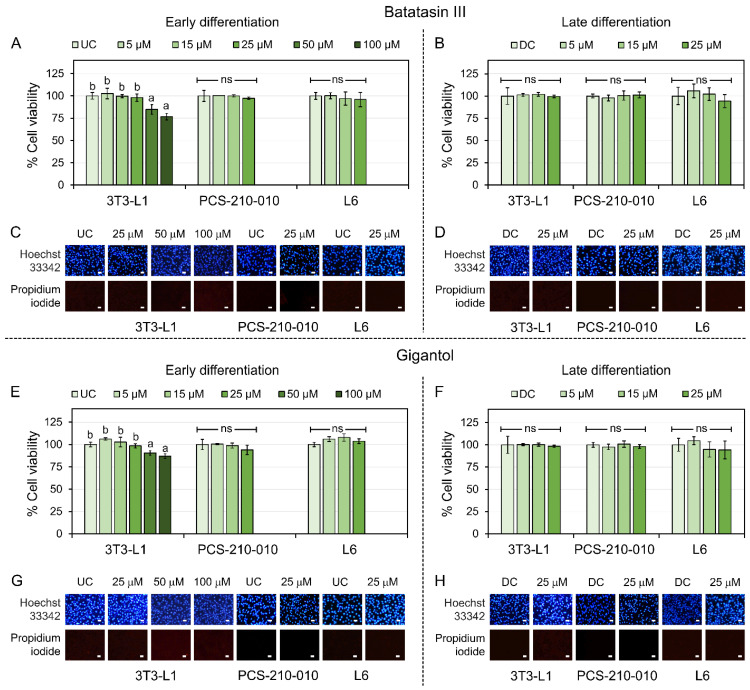
Cytotoxic effects of batatasin III and gigantol in 3T3-L1, PCS-210-010, and L6 cells. The cytotoxic effects of batatasin III and gigantol were assessed in 3T3-L1, PCS-210-010, and L6 cells during early (**A**,**C**,**E**,**G**) and late (**B**,**D**,**F**,**H**) differentiation stages. Cells were treated for 48 h with varying concentrations of batatasin III (**A**–**D**) or gigantol (**E**–**H**) (see Figure S1 for all concentrations tested). Cell viability was determined using the MTT assay (**A**,**B**,**E**,**F**), and the mode of cell death was analyzed through dual nuclear staining with Hoechst 33342 and propidium iodide (**C**,**D**,**G**,**H**). Undifferentiated (UC) and differentiated (DC) cells treated with vehicle (0.5% (*v*/*v*) dimethyl sulfoxide) served as controls. The results are represented as means ± SDs from three independent experiments. Lowercase letters represent statistically significant differences among means within the same experimental condition, whereas ’ns’ indicates no significant statistical difference. Statistical analysis was determined by one-way ANOVA with Tukey’s post hoc test at α = 0.05. Representative images were randomly selected from at least three independent experiments (scale bars = 50 μm).

Batatasin III and gigantol, two phytochemicals derived from *D. venustum* orchid plant materials (Figure 1), were identified and characterized based on MS and NMR spectroscopic data as previously reported [27]. The cytotoxic effects of these compounds were evaluated in mouse 3T3-L1, human PCS-210-010, and rat skeletal muscle L6 cell lines (Figure 2). Concentrations up to 25 μM demonstrated no significant cytotoxicity in 3T3-L1 cells during early differentiation (Figure 2A,E). Similarly, no adverse effects on cell viability were observed in PCS-210-010 and L6 cells during early or late differentiation stages (Figure 2A,B,E,F). Results from the MTT assay (Figure 2A,B,E,F) aligned with findings from the dual nuclear staining method using Hoechst 33342 and propidium iodide (Figure 2C,D,G,H and Figure S1). Consequently, concentrations ≤ 25 μM were deemed non-cytotoxic and used in subsequent experiments.

**Figure 3:**
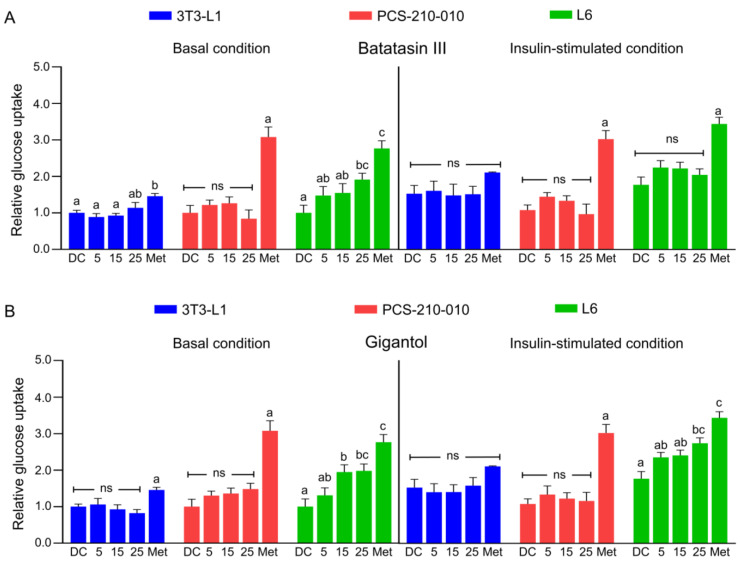
Cellular glucose uptake after treatment with batatasin III and gigantol. The effects of batatasin III (**A**) and gigantol (**B**) on glucose uptake were assessed at non-cytotoxic concentrations of 5–25 μM in differentiated 3T3-L1, PCS-210-010, and L6 cells under both basal and insulin-stimulated conditions using a colorimetric assay. Insulin stimulation was achieved by the addition of 100 nM insulin. Differentiated (DC) cells treated with 0.5% (*v*/*v*) dimethyl sulfoxide served as the vehicle control, while cells treated with 1 mM metformin (Met) served as the positive control. Results are presented as means ± SEMs from at least three independent experiments. Lowercase letters indicate statistically significant differences among means within the same experimental condition, whereas ’ns’ indicates no significant statistical difference. Statistical analysis was performed using one-way ANOVA followed by Tukey’s post hoc test at *α* = 0.05.

The effects of batatasin III and gigantol on cellular glucose uptake were assessed using a colorimetric assay (Figure 3). Batatasin III modestly enhanced basal glucose uptake in 3T3-L1 adipocytes, with a more pronounced effect observed in L6 myotubes under similar conditions (Figure 3A). However, under insulin-stimulated conditions, batatasin III showed no significant impact on glucose uptake across cell types (Figure 3A). Conversely, gigantol significantly increased glucose uptake in L6 myotubes under both basal and insulin-stimulated conditions (Figure 3B). In adipocytes, glucose uptake profiles were consistent across species, though human adipocytes exhibited greater metformin sensitivity compared to mouse adipocytes (Figure 3).

**Figure 4:**
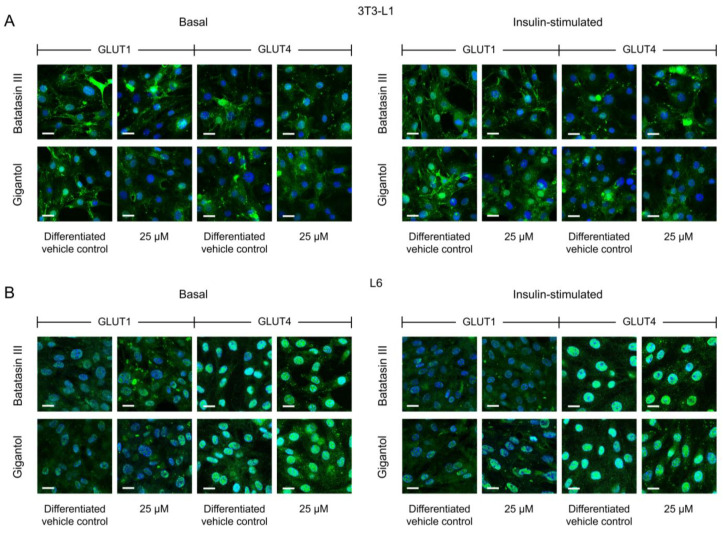
GLUT membrane translocation in 3T3-L1 and L6 cells treated with batatasin III and gigantol. Immunofluorescence imaging was used to assess the localization of GLUT1 and GLUT4 in differentiated 3T3-L1 (**A**) and L6 (**B**) cells treated with non-cytotoxic concentrations of batatasin III and gigantol (up to 25 μM) for 48 h under both basal and insulin-stimulated conditions (refer to Figure S2 for all concentrations tested). Cells treated with 0.5% (*v*/*v*) dimethyl sulfoxide served as vehicle controls. Specific antibodies conjugated to green fluorescence were used to probe GLUT1 and GLUT4, while nuclei were counterstained with Hoechst 33342 (blue fluorescence), illustrating the translocation of GLUT1 and GLUT4 from intracellular compartments to the cell membrane. Images were acquired using a confocal microscope at 20× magnification (scale bar = 20 μm).

To investigate the underlying mechanisms by which batatasin III and gigantol influence cellular glucose uptake, fluorescence microscopy and protein expression analyses were employed. Microscopic observations (Figure 4 and Figure S2) revealed that both compounds impacted GLUT translocation. Notably, these effects were more pronounced at the highest tested concentration of 25 μM, particularly with gigantol treatment, irrespective of insulin stimulation (Figure 4). The observed effects varied between GLUT isoforms (GLUT1 and GLUT4) and cell types (3T3-L1 adipocytes and L6 myotubes). Specifically, GLUT4 translocation was more prominently affected than GLUT1 translocation. Furthermore, gigantol treatments consistently resulted in reduced GLUT membrane localization in 3T3-L1 adipocytes but enhanced localization in L6 myotubes (Figure 4A,B).

**Figure 5:**
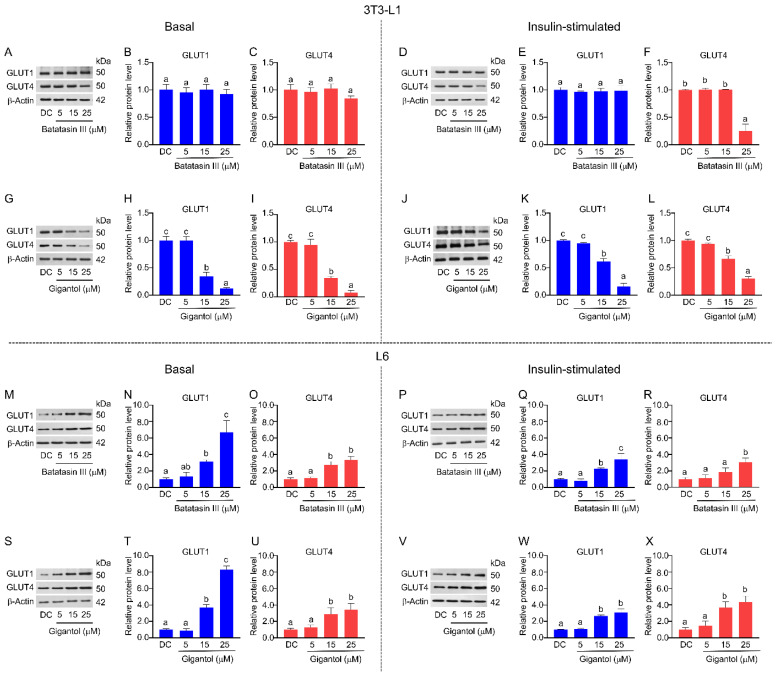
Effects of batatasin III and gigantol on GLUT expression in 3T3-L1 and L6 cells. Western blot analysis was performed to evaluate the expression levels of GLUT1 and GLUT4 in 3T3-L1 adipocytes (**A**–**L**) and L6 myotubes (**M**–**X**) treated with batatasin III or gigantol at non-cytotoxic concentrations (5–25 μM) under basal (**A**–**C**,**G**–**I**,**M**–**O**,**S**–**U**) and insulin-stimulated (**D**–**F**,**J**–**L**,**P**–**R**,**V**–**X**) conditions for 48 h. Insulin stimulation was achieved by the addition of 100 nM insulin. Protein levels were quantified using ImageJ software (Java 1.8.0_172) and normalized to β-actin. Numerical data are presented as means ± SDs from three independent experiments. Lowercase letters indicate statistically significant differences among means within the same experimental condition. Statistical analysis was conducted using one-way ANOVA followed by Tukey’s post hoc test at *α* = 0.05.

Protein expression analysis further demonstrated contrasting effects of the two compounds (Figure 5). Batatasin III had minimal impact on GLUT1 and GLUT4 expression in 3T3-L1 adipocytes, except at 25 μM under insulin-stimulated conditions, where a modest increase was observed (Figure 5A–F). In contrast, gigantol significantly downregulated GLUT1 and GLUT4 expression in 3T3-L1 adipocytes, starting at concentrations as low as 15 μM (Figure 5G–L). In L6 myotubes, both compounds notably upregulated GLUT1 and GLUT4 expression under basal and insulin-stimulated conditions (Figure 5M–X). GLUT1 exhibited particularly high sensitivity to both phytochemicals in basal conditions, with its expression levels increasing up to eightfold compared to vehicle-treated control cells (Figure 5M–O,S–U). Under insulin-stimulated conditions, gigantol at 15 μM induced a more substantial increase in GLUT4 expression than batatasin III did at the same concentration (Figure 5R,X).

### 3.2. Differential Effects on Lipid Metabolism and Adipocyte Differentiation

**Figure 6:**
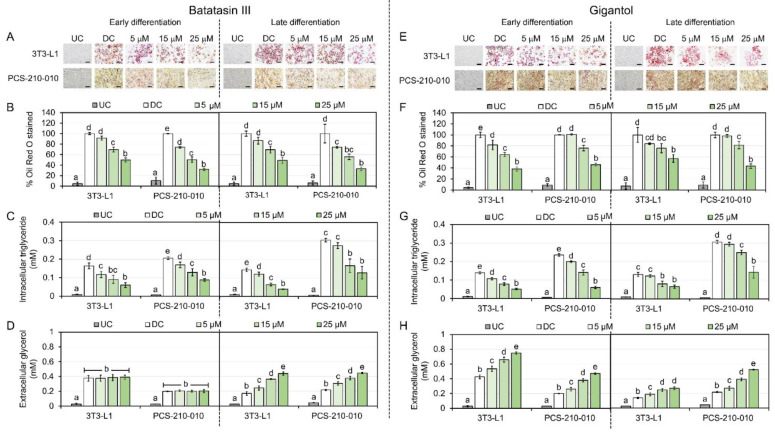
Lipid metabolism effects of batatasin III and gigantol on mouse 3T3-L1 and human PCS-210-010 adipocytic cells. Cells were treated with non-cytotoxic concentrations (5–25 μM) of batatasin III or gigantol for 48 h before assessing cellular lipid droplet accumulation with Oil Red O staining (**A**,**B**,**E**,**F**), intracellular triglyceride levels (**C**,**G**), and extracellular glycerol contents (**D**,**H**). Undifferentiated (UC) and differentiated (DC) cells treated with vehicle (0.5% (*v*/*v*) dimethyl sulfoxide) served as controls. Representative Oil Red O-stained images were embedded with scale bars of 50 μm (**A**,**E**). Results are presented as means ± SDs from three independent experiments. Statistically significant differences among means within the same condition are indicated by lowercase letters. Statistical analysis was performed using one-way ANOVA followed by Tukey’s post hoc test at *α* = 0.05.

The effects of batatasin III and gigantol on lipid metabolism were investigated using Oil Red O staining and measurements of intracellular triglycerides and extracellular glycerol (Figure 6). Both compounds reduced lipid droplet accumulation during early and late differentiation stages in 3T3-L1 and PCS-210-010 cells (Figure 6A,B,E,F), with significant reductions observed at 15–25 μM (Figure 6B,F). Triglyceride levels decreased in both cell lines (Figure 6C,G), while extracellular glycerol levels increased with gigantol but remained unchanged with batatasin III during early differentiation (Figure 6D,H).

### 3.3. Underlying Molecular Mechanisms of Batatasin III and Gigantol in Modulating Lipid Metabolism and Adipocyte Differentiation

**Figure 7:**
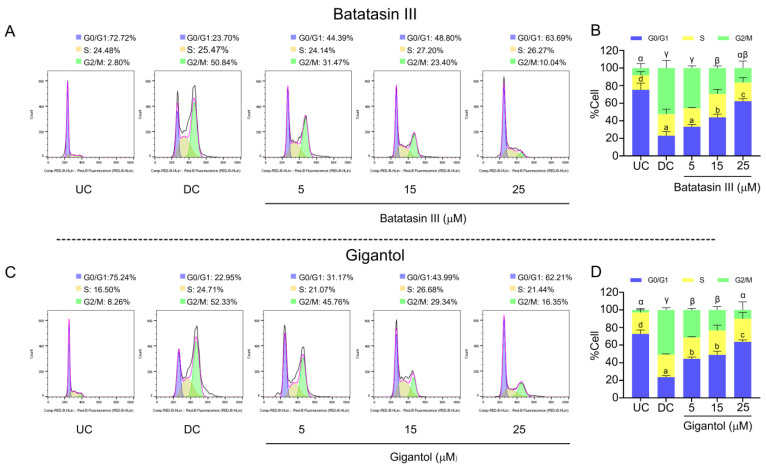
Cell cycle progression in mouse 3T3-L1 pre-adipocytes treated with batatasin III and gigantol. Cell cycle analyses using flow cytometry were conducted after treating 3T3-L1 pre-adipocytic cells treated with varying non-cytotoxic concentrations of batatasin III (**A**,**B**) or gigantol (**C**,**D**) during early differentiation for 24 h. The cell cycle was divided into G0/G1, S, and G2/M phases. Undifferentiated (UC) and differentiated (DC) cells treated with vehicle (0.5% (*v*/*v*) dimethyl sulfoxide) served as controls. Results are presented as means ± SDs from three independent experiments. Statistically significant differences among means within the same condition are indicated by lowercase letters (G0/G1 phase) or Greek letters (G2/M phase). Statistical analysis was performed using one-way ANOVA followed by Tukey’s post hoc test at *α* = 0.05.

To elucidate the mechanisms through which batatasin III and gigantol influence lipid metabolism and adipocyte differentiation, key biological events and the expression of relevant proteins were evaluated. Adipocyte differentiation requires pre-adipocytic cells to undergo several rounds of proliferation, a process known as “mitotic clonal expansion (MCE)” [26,29]. MCE progression was analyzed using flow cytometry to assess cell cycle dynamics (Figure 7). Both compounds induced cell cycle arrest at the G0/G1 phase, reducing the proportion of cells progressing to the G2/M phase (Figure 7A–D). Although the effects were similar between the compounds, gigantol demonstrated stronger activity at the lowest tested concentration (5 μM) (Figure 7B,D).

**Figure 8:**
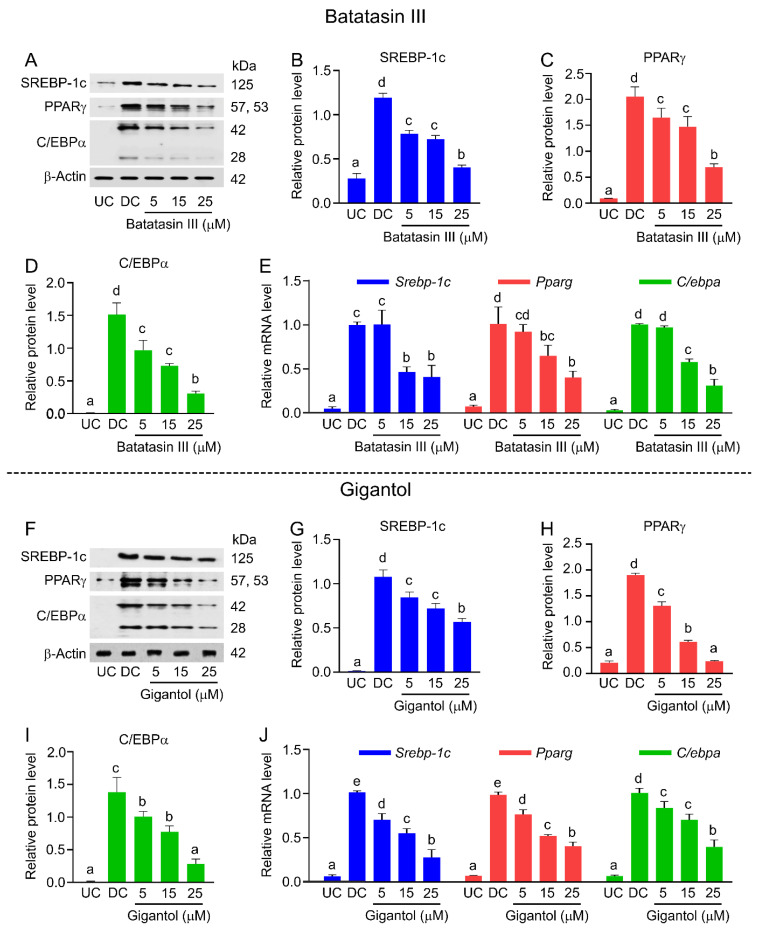
Effects of batatasin III and gigantol on transcription factors in adipocyte differentiation. Protein and gene expression levels of key transcription factors (SREBP-1c, PPARγ, and C/EBPα) in 3T3-L1 cells treated with non-cytotoxic concentrations (5–25 μM) of batatasin III (**A**–**E**) or gigantol (**F**–**J**) during early differentiation for 48 h were assessed using Western blot analysis (**A**–**D**,**F**–**I**) and RT-qPCR (**E**,**J**), respectively. Undifferentiated (UC) and differentiated (DC) cells treated with vehicle (0.5% (*v*/*v*) dimethyl sulfoxide) served as controls. Protein levels were quantified using ImageJ software and normalized to β-actin. Numerical data are presented as means ± SDs from three independent experiments. Lowercase letters indicate statistically significant differences among means within the same experimental condition. Statistical analysis was conducted using one-way ANOVA followed by Tukey’s post hoc test at *α* = 0.05.

In addition to MCE, the expressions of key transcription factors and associated signaling pathways during early adipocyte differentiation were examined [26,30,31,32]. Western blotting (Figure 8A–D,F–I) and RT-qPCR (Figure 8E,J) revealed that both compounds downregulated the expression of sterol regulatory element-binding protein-1c (SREBP-1c), peroxisome proliferator-activated receptor γ (PPARγ), and CCAAT/enhancer-binding protein α (C/EBPα) in a dose-dependent manner. Notably, gigantol at 5 μM significantly reduced transcriptional levels of these factors (*Srebp-1c*, *Pparg*, and *C/ebpa*), whereas batatasin III had negligible effects at the same concentration (Figure 8E,J).

**Figure 9:**
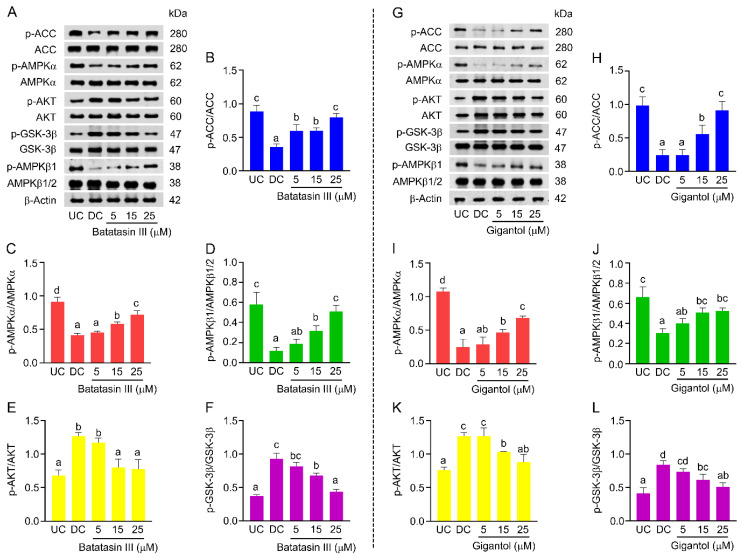
Impacts of batatasin III and gigantol on adipogenesis-mediated signaling pathways. The activation (phosphorylation denoted with prefix p-) levels of some signaling pathways (AMPK-ACC and AKT/GSK-3 β) triggered during early differentiation of 3T3-L1 cells treated with non-cytotoxic concentrations (5–25 μM) of batatasin III (**A**–**F**) or gigantol (**G**–**L**) for 48 h were assessed using Western blot analysis. Undifferentiated (UC) and differentiated (DC) cells treated with vehicle (0.5% (*v*/*v*) dimethyl sulfoxide) served as controls. Protein levels were quantified using ImageJ software and normalized to β-actin. Numerical data are presented as means ± SDs from three independent experiments. Lowercase letters indicate statistically significant differences among means within the same experimental condition. Statistical analysis was conducted using one-way ANOVA followed by Tukey’s post hoc test at *α* = 0.05.

The impact of these bibenzyl compounds on signaling pathways associated with adipogenesis, including the AMP-activated protein kinase-acetyl-CoA carboxylase (AMPK-ACC) and protein kinase B/glycogen synthase kinase-3β (AKT/GSK-3β) pathways, was also investigated (Figure 9). During early adipocyte differentiation, the AMPK-ACC pathway is typically downregulated through phosphorylation, while the AKT/GSK-3β pathway is activated [26,33,34]. Western blot analysis demonstrated that both batatasin III (Figure 9A–F) and gigantol (Figure 9G–L) modulated these pathways by activating AMPK-ACC and deactivating AKT/GSK-3β in 3T3-L1 cells. Both compounds exhibited similar trends in pathway modulation.

**Figure 10:**
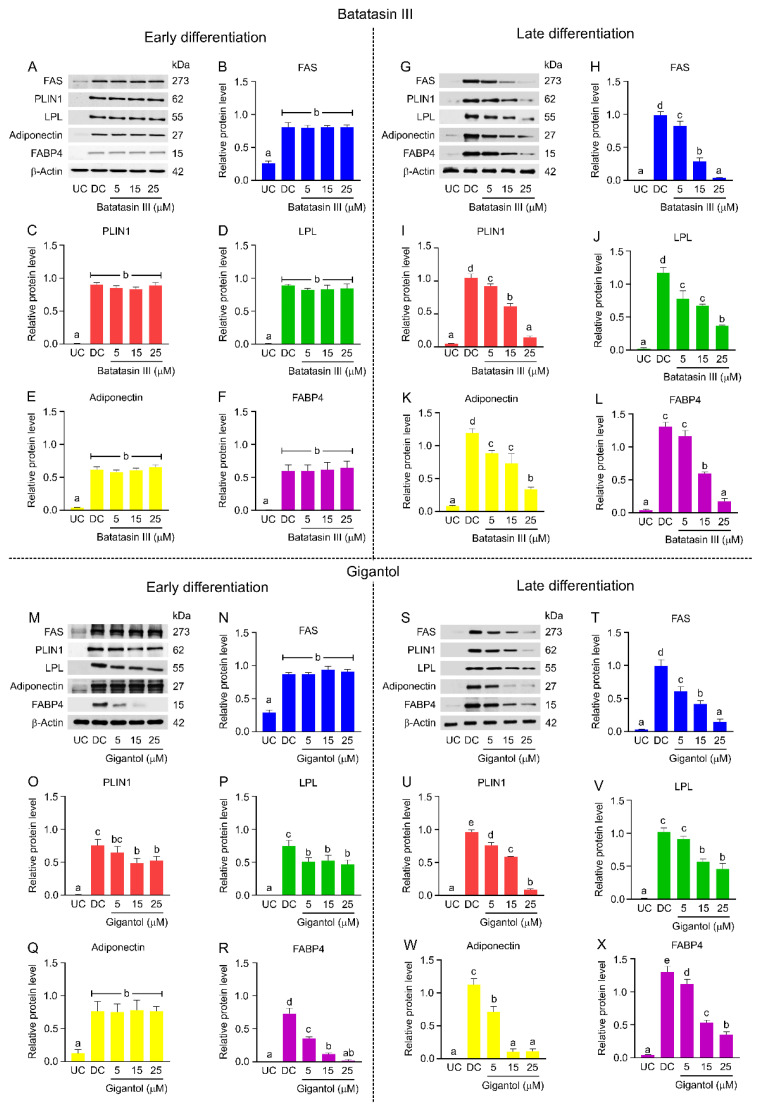
Effects of batatasin III and gigantol on lipogenic protein expression. The levels of some lipogenic proteins (FAS, PLIN1, LPL, adiponectin, and FABP4) expressed during early (**A**–**F**,**M**–**R**) and late (**G**–**L**,**S**–**X**) differentiation of 3T3-L1 cells treated with non-cytotoxic concentrations (5–25 μM) of batatasin III (**A**–**L**) or gigantol (**M**–**X**) for 48 h were assessed using Western blot analysis. Undifferentiated (UC) and differentiated (DC) cells treated with vehicle (0.5% (*v*/*v*) dimethyl sulfoxide) served as controls. Protein levels were quantified using ImageJ software and normalized to β-actin. Numerical data are presented as means ± SDs from three independent experiments. Lowercase letters indicate statistically significant differences among means within the same experimental condition. Statistical analysis was conducted using one-way ANOVA followed by Tukey’s post hoc test at *α* = 0.05.

The expression of lipogenic proteins, including fatty acid synthase (FAS), perilipin 1 (PLIN1), lipoprotein lipase (LPL), adiponectin, and fatty acid-binding protein 4 (FABP4), was further analyzed using Western blotting (Figure 10) [35,36,37,38,39]. Batatasin III did not affect lipogenic protein levels during early differentiation (Figure 10A–F) but significantly downregulated their expression during late differentiation (Figure 10G–L). Conversely, gigantol reduced PLIN1, LPL, and FABP4 expression during early differentiation (Figure 10M,O,P,R) and downregulated all tested lipogenic proteins during late differentiation (Figure 10S–X). Among the proteins affected during early differentiation, FABP4 displayed the most pronounced dose-dependent response to gigantol.

**Figure 11:**
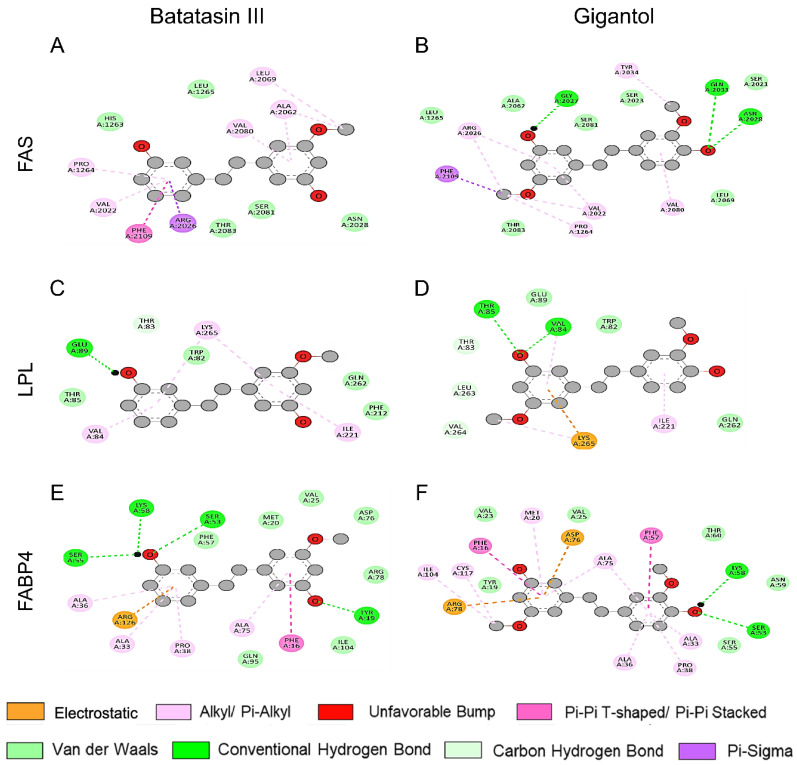
Molecular docking analysis depicting interactions between lipogenic proteins and batatasin III or gigantol. The two–dimensional interaction diagrams demonstrate the optimal docking poses of batatasin III (**A**,**C**,**E**) and gigantol (**B**,**D**,**F**) with selected lipogenic proteins (FAS (**A**,**B**), LPL (**C**,**D**), and FABP4 (**E**,**F**)). These diagrams highlight the molecular interactions and binding sites, guiding insights into the mechanisms by which these compounds engage with the target proteins.

To further investigate the distinct roles of batatasin III and gigantol in modulating cellular lipid metabolism, their intermolecular interactions with key lipogenic proteins, including FAS, LPL, and FABP4, were analyzed using in silico molecular docking (Figure 11). Both compounds exhibited similar binding interactions with amino acid residues in the FAS structure (Figure 11A,B), predominantly mediated by hydrophobic interactions and van der Waals (vdW) forces. However, gigantol demonstrated enhanced binding affinity through the formation of three additional hydrogen bonds and its methoxy group at the 5′-carbon position establishing four distinct interactions with key FAS residues, including Pro1264, Phe2109, Val2022, and Arg2026 (Figure 11B). These interactions were also observed in batatasin III-FAS docking simulations (Figure 11A). Moreover, the methoxy group at the 3-carbon position of gigantol was predicted to interact with Tyr2034 in FAS, while its hydroxyl group at the 3′-carbon position formed hydrogen bonds with Gly2027. Additional stabilizing interactions included hydrogen bonding between the hydroxyl group at the 4-carbon position of gigantol and the FAS residues Asn2028 and Gln2031.

The molecular docking analysis of both compounds with LPL revealed similar binding patterns (Figure 11C,D). Two additional amino acids weakly bind to gigantol, including Leu263 and Val264 mediated by vdW forces. Similarly, docking studies with FABP4 indicated overlapping binding sites for batatasin III and gigantol (Figure 11E,F). Specifically, gigantol demonstrated enhanced binding affinity through additional interactions with Val23, Asn59, and Thr60 via vdW forces, as well as an extra hydrophobic interaction with Cys117 in FABP4 (Figure 11F). The methoxy group at the 5′-carbon position of gigantol further contributed to its binding stability by engaging in hydrophobic interactions with Ile104 and Cys117, which were absent in the batatasin III docking model. Conversely, the hydroxyl group at the 3′-carbon position of batatasin III established hydrogen bonds with Ser53, Ser55, and Lys58 residues in FABP4 (Figure 11E). Additionally, a unique electrostatic interaction with Arg126 was observed exclusively in the docking of batatasin III with FABP4.

## 4. Discussion

Bibenzyl compounds are widely distributed in orchids, particularly within the *Dendrobium* genus, where they are believed to play a role in defense mechanisms and pigmentation regulation [40]. Recent research has highlighted the pharmacological potential of bibenzyl derivatives in managing metabolic disorders, such as obesity and type 2 diabetes, primarily due to their anti-inflammatory, antioxidant, and insulin-sensitizing properties [18,41,42]. Structurally, bibenzyls are a diverse class of secondary metabolites that can be described as ethane derivatives, where each carbon atom is bonded to a phenyl group. Their biological activity is largely influenced by structural modifications, especially the nature and position of arene substitutions [12,18]. Our study further demonstrated that structural diversification of orchid-derived bibenzyls, specifically batatasin III and gigantol, significantly affects their bioactivity in modulating cellular glucose uptake and lipid metabolism, highlighting the crucial role of arene substitutions in their pharmacological effects.

The role of arene substitution in the cytotoxicity of batatasin III and gigantol remains inconclusive. Both bibenzyl compounds exhibited no detectable cytotoxic effects across all tested cell lines at concentrations up to 25 μM despite variations in their methoxy and hydroxyl substitution patterns. Specifically, gigantol possesses two methoxy groups at meta positions (3- and 5′-carbon), whereas batatasin III contains a single methoxy group at the 3-carbon position. In comparison, when 3T3-L1 cells were treated with other bibenzyl derivatives featuring two methoxy substitutions, 4,5,4′-trihydroxy-3,3′-dimethoxybibenzyl (TDB) displayed subtoxic effects at concentrations ≤5 μM [26], while 3,4-dihydroxy-5,4′-dimethoxybibenzyl (DDB) exhibited reduced cytotoxicity at concentrations ≤10 μM [25]. These findings suggest that the number and positioning of hydroxyl and methoxy groups may influence cytotoxic activity, with TDB containing three hydroxyl groups, whereas the other bibenzyl derivatives contain only two. This observation aligns with a previous study demonstrating that the number and position of methoxy and hydroxyl groups in the arene substitution contribute to variations in phytotoxicity among natural and synthetic bibenzyl compounds [12].

In a preliminary glucose uptake assay, batatasin III demonstrated limited efficacy in enhancing glucose uptake in L6 myotubes under insulin-stimulated conditions compared to gigantol treatment. Previous studies have evaluated the ability of various bibenzyl compounds, including batatasin III, gigantol, aloifol I, dendrosinen B, and moscatilin, to stimulate glucose uptake in L6 cells [41,42]. Specifically, batatasin III at 41 μM and aloifol I at 36 μM exhibited a stimulatory effect on glucose uptake in L6 myotubes [41], whereas gigantol (at 3.65, 36.5, and 365 μM) and other bibenzyl derivatives did not enhance glucose uptake [42]. These findings cannot be directly compared to our results, as our experiments were conducted at defined concentrations of 5, 15, and 25 μM, with significant stimulatory effects observed at 25 μM. However, the biological roles of bibenzyl compounds in promoting glucose uptake in myotubes and reducing glucose uptake in adipocytes remain poorly understood.

In this study, we further examined potential underlying mechanisms by assessing the effects of batatasin III and gigantol on GLUT translocation and expression. Both compounds exhibited similar effects on the translocation patterns of GLUT1 and GLUT4, which corresponded with their influence on GLUT1 and GLUT4 expression levels. Notably, gigantol induced a more pronounced downregulation of GLUT1 and GLUT4 expression in 3T3-L1 adipocytes than batatasin III did. This differential response may be attributed to structural variations between the two bibenzyls. Further studies are necessary to elucidate the precise role of these bibenzyl compounds in regulating GLUT transcription and translation, as well as to determine the specific molecular interactions responsible for these variations.

Obesity is a complex metabolic disorder characterized by excessive adipose tissue accumulation, primarily driven by adipocyte hypertrophy and hyperplasia [43]. Adipocyte differentiation, a critical process in adipogenesis, serves as a valuable model for evaluating the anti-obesity potential of emerging pharmaceutical candidates. In this study, we investigated the effects of batatasin III and gigantol on lipid metabolism in adipocytic cells. Both bibenzyls exhibited comparable effects; however, batatasin III did not significantly influence extracellular glycerol levels during the early stages of adipocyte differentiation, indicating potential differences in lipid mobilization mechanisms.

To elucidate the underlying mechanisms governing early adipocyte differentiation, we further assessed key regulatory pathways in 3T3-L1 cells, including MCE, essential transcription factors (SREBP-1c, PPARγ, and C/EBPα), competitive phosphorylation of ACC-AMPK and AKT/GSK-3β pathways, and expression of key lipogenic proteins (FAS, PLIN1, LPL, adiponectin, and FABP4) [26,35,36,37,38]. While both bibenzyls exerted similar effects on these biological events, differences were observed in the regulation of lipogenic proteins, particularly PLIN1, LPL, and FABP4, where gigantol exhibited a more pronounced impact than batatasin III did. A comparable trend was observed with TDB treatment; however, TDB effectively downregulated all examined lipogenic proteins during the early differentiation phase of 3T3-L1 cells [26].

The differential effects of the two bibenzyl compounds on lipogenic proteins are likely influenced by structural diversification, particularly the nature and position of arene substitutions. However, molecular docking analysis revealed that the interactions between batatasin III or gigantol and certain lipogenic proteins did not exhibit significant differences. This finding suggests that variations in bibenzyl structures may primarily impact upstream regulatory mechanisms preceding protein expression and function, such as transcriptional regulation or epigenetic modifications. For instance, differences in glucose uptake affected by batatasin III and gigantol could contribute to alterations in glycerol release and lipogenic protein expression. These observations highlight the need for further investigations to explore the interactions between these bibenzyl compounds and key molecules involved in the pre-translational regulation of lipogenic proteins. A deeper understanding of these molecular mechanisms will be essential to bridge the existing knowledge gap and provide insights into the regulatory roles of bibenzyls in lipid metabolism.

Although our findings provide insights into the metabolic effects of bibenzyl compounds, the study has some limitations. First, our experiments were conducted in vitro, and thus the observed effects may not fully translate to in vivo systems due to differences in metabolic processing, bioavailability, and systemic interactions. Second, while we identified differential effects on GLUT expression and lipid metabolism, the precise molecular targets and signaling pathways remain to be fully elucidated.

To address these limitations, future studies should explore the in vivo pharmacokinetics and bioavailability of these bibenzyls in animal models of obesity and diabetes. Additionally, a deeper mechanistic analysis, including RNA sequencing and chromatin immunoprecipitation assays, could reveal upstream regulatory factors involved in GLUT and lipid metabolism modulation.

## 5. Conclusions

This study provides valuable insights into the structural and functional roles of orchid-derived bibenzyls, particularly batatasin III and gigantol, in metabolic regulation. Both compounds exhibited significant effects on glucose uptake, GLUT translocation, and lipid metabolism, with structural diversification influencing their bioactivity. Gigantol exhibited a stronger influence on the downregulation of GLUT1 and GLUT4 expression in adipocytes and more pronounced effects on key lipogenic proteins than batatasin III did. The observed differences underscore the importance of arene substitutions in dictating the metabolic activities of these bibenzyl derivatives.

Given the growing interest in natural compounds as potential therapeutics for metabolic disorders, further studies are warranted to explore the preclinical efficacy, bioavailability, and pharmacokinetic properties of bibenzyls in obesity and diabetes models. In vivo validation is essential to confirm the metabolic effects observed in vitro and to determine potential systemic interactions. Additionally, structure–activity relationship studies could facilitate the development of optimized bibenzyl derivatives with enhanced potency and specificity.

These findings contribute to the broader understanding of plant-derived bioactive compounds and highlight their potential application in the development of novel metabolic therapeutics.

## Data Availability

Data are contained within the article.

## Supplementary Materials

The following supporting information can be downloaded at: https://www.mdpi.com/article/10.3390/nu17071104/s1, Table S1: List of primers used in this study; Table S2: Three-dimensional coordinates of ligands used for molecular docking analysis; Figure S1: Cytotoxic effects of batatasin III and gigantol in 3T3-L1, PCS-210-010, and L6 cells. The cytotoxic effects of batatasin III and gigantol were assessed in 3T3-L1, PCS-210-010, and L6 cells during early (A and C) and late (B and D) differentiation stages. Cells were treated for 48 h with varying concentrations of batatasin III (A and B) or gigantol (C and D). The mode of cell death was analyzed through dual nuclear staining with Hoechst 33342 and propidium iodide. Undifferentiated (UC) and differentiated (DC) cells treated with vehicle (0.5% (*v*/*v*) dimethyl sulfoxide) served as controls. Representative images were randomly selected from at least three independent experiments (scale bars = 50 μm). Figure S2: GLUT membrane translocation in 3T3-L1 and L6 cells treated with batatasin III and gigantol. Immunofluorescence imaging was used to assess the localization of GLUT1 (A, E, C, G, I, M, K, and O) and GLUT4 (B, F, D, H, J, N, L, and P) in differentiated 3T3-L1 (A-H) and L6 (I-P) cells treated with non-cytotoxic concentrations of batatasin III and gigantol (up to 25 μM) for 48 h under both basal and insulin-stimulated conditions. Cells treated with 0.5% (*v*/*v*) dimethyl sulfoxide served as vehicle controls. Specific antibodies conjugated to green fluorescence were used to probe GLUT1 and GLUT4, while nuclei were counterstained with Hoechst 33342 (blue fluorescence), illustrating the translocation of GLUT1 and GLUT4 from intracellular compartments to the cell membrane. Images were acquired using a confocal microscope at 20× magnification (scale bar = 20 μm).
