# Supplementary material for: Arene Substitutions in Orchid Bibenzyls: Mechanistic Insights into Glucose Uptake and Lipid Metabolism for Targeting Metabolic Disorders
Source: Nutrients. 2025 Mar 21;17(7):1104. doi: 10.3390/nu17071104 (PMC11990513; doi:10.3390/nu17071104)

Supplementary Materials

# Arene Substitutions in Orchid Bibenzyls: Mechanistic Insights into Glucose Uptake and Lipid Metabolism for Targeting Metabolic Disorders

Narawat Nuamnaichati [1], Utid Suriya [2], Hnin Ei Ei Khine [1], Rungroch Sungthong [1], Poon Suwannamai [3], Boonchoo Sritularak [4, 5], Eakachai Prompetchara [6, 7], Chavee Laomeephol [1], Rosa Alduina [8], and Chatchai Chaotham [1, 9, *]

[1] Department of Biochemistry and Microbiology, Faculty of Pharmaceutical Sciences, Chulalongkorn University, Bangkok 10330, Thailand

[2] Department of Biochemistry, Faculty of Science, Mahidol University, Bangkok, 10400, Thailand

[3] Department of Biotechnology, Faculty of Science, Mahidol University, Bangkok, 10400, Thailand

[4] Department of Pharmacognosy and Pharmaceutical Botany, Faculty of Pharmaceutical Sciences, Chulalongkorn University, Bangkok 10330, Thailand

[5] Center of Excellence in Natural Products for Ageing and Chronic Diseases, Faculty of Pharmaceutical Sciences, Chulalongkorn University, Bangkok 10330, Thailand

[6] Department of Laboratory Medicine, Faculty of Medicine, Chulalongkorn University, Bangkok 10330, Thailand

[7] Center of Excellence in Vaccine Research and Development (Chula Vaccine Research Center), Faculty of Medicine, Chulalongkorn University, Bangkok 10330, Thailand

[8] Department of Biological, Chemical and Pharmaceutical Sciences and Technologies (STEBICEF), University of Palermo, Palermo 90128, Italy

[9] Center of Excellence in Preclinical Toxicity and Efficacy Assessment of Medicines and Chemicals, Chulalongkorn University, Bangkok, 10330, Thailand

* Correspondence: cchoatham@gmail.com

**Table S1.** List of primers used in this study.

| Primer | Nucleotide sequence (5' - 3') | Number of nucleotides | ΔG (kcal mol$^{-1}$) | $T_m$ (°C) | $T_a$ (°C) |
|---|---|---|---|---|---|
| PPARγ F | GATTCTCCTRTTGACCCAG | 19 | 1.06 | 51.0 | 55 |
| PPARγ R | GARTGSGAGTGGTCTTCCAT | 20 | -0.95 | 55.7 | 55 |
| C/EBPα F | AGTCGGTGGACAAGAACAGC | 20 | -1.62 | 57.4 | 55 |
| C/EBPα R | GTGTCCAGTTCRCGGCTCA | 19 | 0.92 | 59.0 | 55 |
| SREBP1 F | YTGCMGACCCTGGTGAGTG | 19 | -0.32 | 59.1 | 55 |
| SREBP1 R | GASCGGTAGCGCTTCTCAAT | 20 | -1.74 | 57.4 | 55 |
| β-Actin F | CCACCATGTACCCWGGCATT | 20 | 0.68 | 57.6 | 55 |
| β-Actin R | CGGACTCRTCRTACTCCTGC | 20 | 0.08 | 57.2 | 55 |

**Table S2.** Three-dimensional coordinates of ligands used for molecular docking analysis.

| Ligand [a] | Coordinate | | |
|---|---|---|---|
| | X | Y | Z |
| FAS | 10.561091 | 6.370303 | -33.511758 |
| LPL | 2.501486 | 0.863000 | 102.519514 |
| FABP 4 | -4.274389 | 6.863889 | -19.213944 |

[a] The ligands selected for molecular docking included fatty acid synthase (FAS, PDB ID: 4PIV), lipoprotein lipase (LPL, PDB ID:6OB0), and fatty acid binding protein 4 (FABP4, PDB ID: 4NNT). Their three-dimensional structures were obtained from the Protein Data Bank (PDB).

**Figure S1.** Cytotoxic effects of batatasin III and gigantol in 3T3-L1, PCS-210-010, and L6 cells. The cytotoxic effects of batatasin III and gigantol were assessed in 3T3-L1, PCS-210-010, and L6 cells during early (A and C) and late (B and D) differentiation stages. Cells were treated for 48 h with varying concentrations of batatasin III (A and B) or gigantol (C and D). The mode of cell death was analyzed through dual nuclear staining with Hoechst 33342 and propidium iodide. Undifferentiated (UC) and differentiated (DC) cells treated with vehicle (0.5% (v/v) dimethyl sulfoxide) served as controls. Representative images were randomly selected from at least three independent experiments (scale bars = 50 μm).

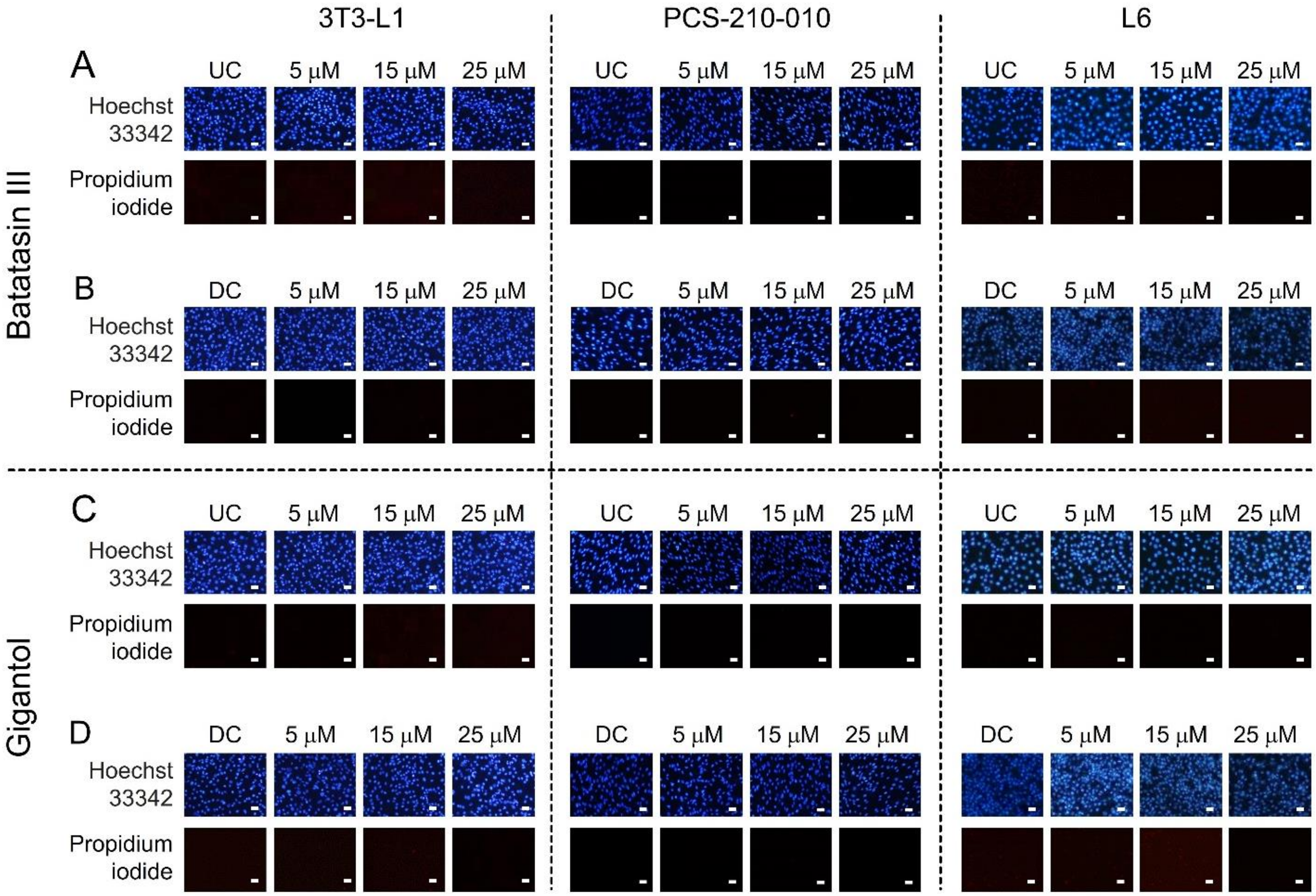

**Figure S2.** GLUT membrane translocation in 3T3-L1 and L6 cells treated with batatasin III and gigantol. Immunofluorescence imaging was used to assess the localization of GLUT1 (A, E, C, G, I, M, K, and O) and GLUT4 (B, F, D, H, J, N, L, and P) in differentiated 3T3-L1 (A-H) and L6 (I-P) cells treated with non-cytotoxic concentrations of batatasin III and gigantol (up to 25 μM) for 48 h under both basal and insulin-stimulated conditions. Cells treated with 0.5% (v/v) dimethyl sulfoxide served as vehicle controls. Specific antibodies conjugated to green fluorescence were used to probe GLUT1 and GLUT4, while nuclei were counterstained with Hoechst 33342 (blue fluorescence), illustrating the translocation of GLUT1 and GLUT4 from intracellular compartments to the cell membrane. Images were acquired using a confocal microscope at 20X magnification (scale bar = 20 μm).

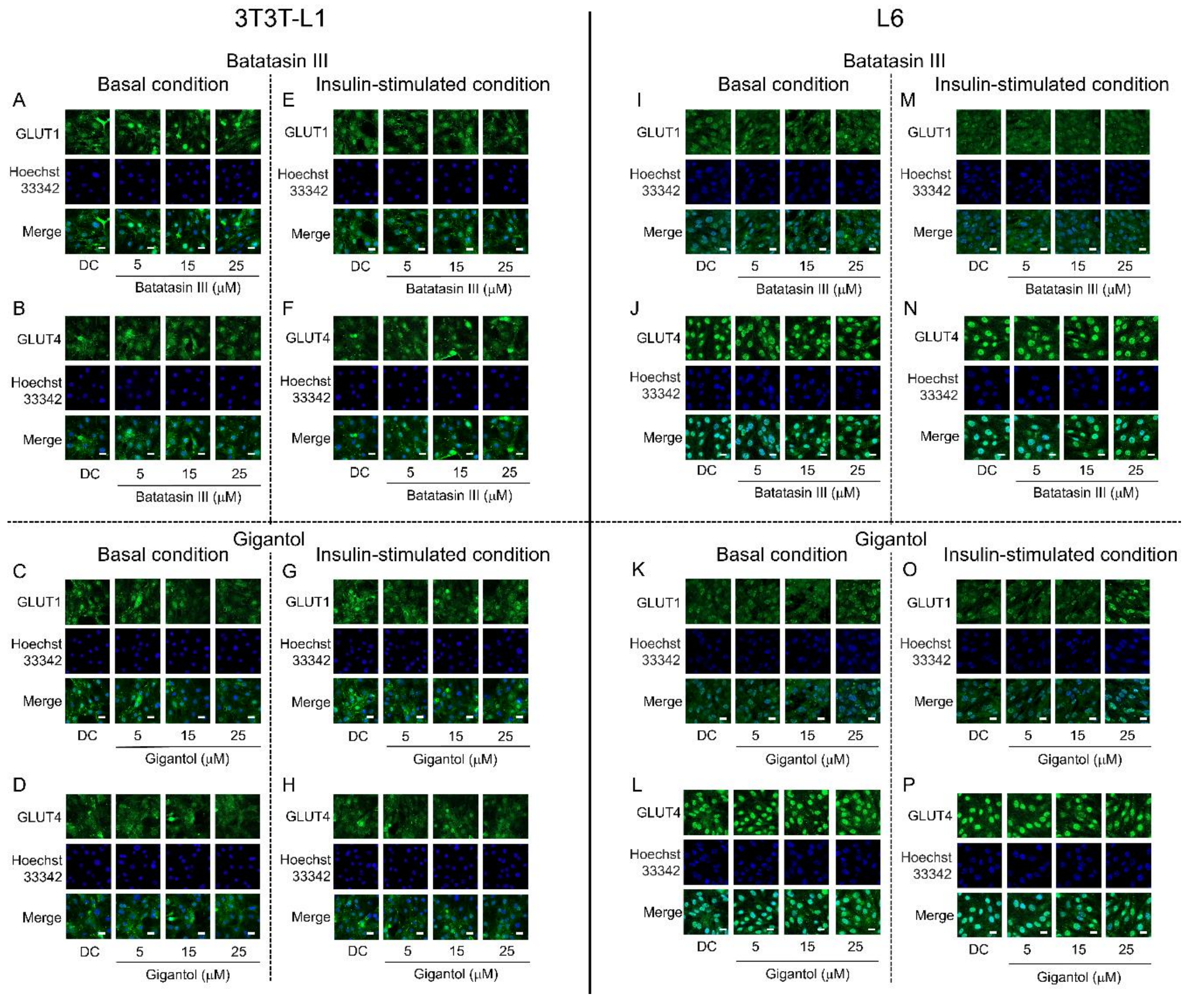

Supplement: Supplementary file 1 [file nutrients-17-01104-s001.zip › nutrients-3540907-supplementary.pdf]
